# Ointments and Oleates

**Published:** 1891-03

**Authors:** 


Ointments and Oleates, Especially in Diseases of the Skin. By John V. Shoemaker, A. M., M. D., Professor of Materia Medica, Pharmacology, and Clinical Medicine, and Clinical Professor of Diseases of the Skin in the Medico-Chirurgical College of Philadelphia; Physician to the Medico-Chirurgical Hospital, etc. Second edition, revised and enlarged. No. 6 in the Physician's and Student's Ready Reference Series. Philadelphia and London: F. A. Davis. 1890. Price, $1.50 net.

This is one of the most useful of the recent additions to the literature of materia medica and pharmacy. Ointments and oleates must ever play an important part in the treatment of disease, and it is well to have their preparation and mode of application grouped in a convenient form for reference. Dr. Shoemaker has done his work admirably in this little treatise, and it should have a ready sale. The price is reasonable, and the make-up of the book is handsome in every way. This second edition contains additions and revisions that fetches it forward to the latest date. The oleates are given a prominence that they deserve, and full directions are given for their combination with appropriate drugs and remedies. We repeat that this is a work that has been well prepared, and is deserving a place on the book-shelves of every progressive physician.

